# Draft genome sequence of *Streptomyces* sp. strain ICN988, isolated from *Gorgonia* sp.

**DOI:** 10.1128/MRA.00285-23

**Published:** 2023-08-18

**Authors:** Sangeetha Nallathambi, Albert Joshua Sam, Samuel Gnana Prakash Vincent

**Affiliations:** 1 Centre for Marine Science and Technology (CMST), Manonmaniam Sundaranar University, Rajakkamangalam, Tamil Nadu, India; Montana State University, Bozeman, Montana, USA

**Keywords:** whole genome sequence, marine actinomycetes

## Abstract

This study elucidates the draft genomic sequence of *Streptomyces* sp. strain ICN988, an actinomycete isolated from *Gorgonia*. The assembled genome consists of 6,122,654 bp with a GC content of 73%. A comprehensive analysis revealed 19 biosynthetic gene clusters.

## ANNOUNCEMENT

Actinomycetes produce many secondary metabolites, including antibiotics, antitumor agents, and immunosuppressive compounds, and are accountable for over half of the antibiotics used clinically (1). Marine actinomycetes can produce diverse compounds due to unique environmental habitats (2, 3). Compounds from marine actinomycetes are important for developing new drugs that benefit human health and industry (4, 5).

The actinomycete *Streptomyces* sp. strain ICN988 was isolated from a *Gorgonia* sea-fan by-catch collected from Colachel coastal area, Tamil Nadu, India (8°10'22.0"N 77°14'55.2"E) on 28 December 2018. Initially washed with seawater to remove surface deposits, *Gorgonia*’s holdfast was scraped aseptically, and the sand was suspended in 2% NaCl to prepare serial dilutions, then spread on Actinomycete Isolation Media (AIM) agar plates (supplemented with filter-sterilized 10 µg/mL nystatin and 50 µg/mL cycloheximide). *Streptomyces* sp. strain ICN988 (S5K) was one of the actinomycetes found after 7 days of incubation at 30°C. Genomic DNA was isolated from 5-day-old axenic ICN988 aerial mycelia using the NucleoSpin Tissue Kit (Macherey-Nagel). The 16S rRNA was PCR amplified with 63F forward primer and 1387R reverse primer. The actinomycete’s 16S rRNA gene (GenBank: OP585661) was closely related (98.34% similarity) to *Streptomyces ardesiacus* NBRC 15402 (GenBank: NR_112454) via NCBI-BLAST and (96.60% similarity) to *Streptomyces coelicoflavus* NBRC 15399 (GenBank: AB184650) via EzBioCloud database (6).

The genomic DNA was extracted from a 7-day-old AIM agar plate culture using HiPurA *Streptomyces* DNA Purification Kit (HiMedia Laboratories, India). Sequence libraries were created using Nextera XT DNA Library Preparation Kit (Illumina). The whole-genome sequencing was obtained using Illumina HiSeq 4000 System (paired-end 150 bp). A total of 18,237,528 bp reads were obtained with 2,753,866,728 total read bases. The GC content was 71.01%, and Q30 was 88.73%. The tools such as Trimmomatic (v 0.36) for adapter trimming (7), FastQC (v0.11.5) for quality check (8), and SPAdes v3.15.0 for read assembly (9) utilized default parameters. After assembly, the genome had 8,344,193 bp in 619 scaffolds. The scaffold N_50_ was 22,387 bp; the GC content was 72%. The assembly completeness with BUSCO (v3.0.2) (10) revealed 120 (96.77%) complete and single-copy BUSCOs, 4 (3.23 %) complete and duplicated BUSCOs, and no fragmented or missing BUSCOs. The genome annotation with NCBI Prokaryotic Genome Annotation Pipeline v6.3 (11) identified 7,718 total genes (CDS), 7,573 protein-coding genes, 9 rRNA genes, 68 tRNAs, three ncRNAs, and 145 pseudogenes.

Through antiSMASH v7.0.0beta1-67b538a9 (12, 13), 30 classes of biosynthetic gene clusters, including terpenes, nonribosomal peptide synthetase, polyketides, ribosomally produced, and post-translationally modified peptides, were found within 37 regions in varied percentages of similarity. Cluster identity was 100% for sapB, germicidin, coelichelin, albaflavenone, hopene, flaviolin, alkyl-resorcinol, ectoine, geosmin, and melanin. In-depth gene cluster mining is necessary for discovering pharmacologically significant novel secondary metabolites. The *Streptomyces* sp. strain ICN988 was antagonistic to methicillin-resistant *Staphylococcus aureus* ATCC 33591 in agar-plug and metabolic extract disc diffusion assays.

## Data Availability

Sequencing data is deposited in GenBank under BioProject accession PRJNA887201, BioSample accession SAMN31156303, and GenBank accession NZ_JAOVZD000000000. Raw sequences were deposited in SRA with accession SRR21823496.
